# Prognostic Impact of Sarcopenia in Patients with Metastatic Hormone-Sensitive Prostate Cancer

**DOI:** 10.3390/cancers13246345

**Published:** 2021-12-17

**Authors:** Ji Hyun Lee, Byul A Jee, Jae-Hun Kim, Hoyoung Bae, Jae Hoon Chung, Wan Song, Hyun Hwan Sung, Hwang Gyun Jeon, Byong Chang Jeong, Seong Il Seo, Seong Soo Jeon, Hyun Moo Lee, Se Hoon Park, Minyong Kang

**Affiliations:** 1 Department of Radiology, Samsung Medical Center, Sungkyunkwan University School of Medicine, Seoul 06351, Korea; carrot302@hotmail.com (J.H.L.); jaehun.kim78@gmail.com (J.-H.K.); 2 Department of Urology, Samsung Medical Center, Sungkyunkwan University School of Medicine, Seoul 06351, Korea; astherjee@hanmail.net (B.A.J.); ho.bae@samsung.com (H.B.); jaehoontasker.chung@samsung.com (J.H.C.); wan.song@samsung.com (W.S.); hyunhwan.sung@samsung.com (H.H.S.); hwanggyun.jeon@samsung.com (H.G.J.); bc2.jung@samsung.com (B.C.J.); seongil.seo@samsung.com (S.I.S.); seongsoo.jeon@samsung.com (S.S.J.); hyunmoo.lee@samsung.com (H.M.L.); 3 Division of Hematology-Oncology, Department of Internal Medicine, Samsung Medical Center, Sungkyunkwan University School of Medicine, Seoul 06351, Korea; sh1767.park@samsung.com; 4 Samsung Genome Institute, Samsung Medical Center, Seoul 06351, Korea; 5 Department of Health Sciences and Technology, SAIHST, Sungkyunkwan University, Seoul 06351, Korea; 6 Department of Digital Health, SAIHST, Sungkyunkwan University, Seoul 06355, Korea

**Keywords:** prostate cancer, sarcopenia, prognosticator, docetaxel, abiraterone acetate

## Abstract

**Simple Summary:**

As sarcopenia is recognized as a poor prognostic factor in various type of cancers, we hypothesized that sarcopenia may also have adverse impact in patients with metastatic hormone-sensitive prostate cancer (mHSPC). In this study, we found that sarcopenia is an independent prognostic factor for poor failure-free survival and time to prostate-specific antigen progression in patients with mHSPC who receive early docetaxel or abiraterone acetate treatment. In addition, we performed RNA sequencing of primary tumors to further understand the biological perspective of the presence of sarcopenia in mHSPC. Transcriptomic differences were found between primary tumors with and without sarcopenia, which may have a potential to link between sarcopenia and poor clinical outcomes in these patients.

**Abstract:**

The clinical value of sarcopenia has not been determined yet in metastatic hormone-sensitive prostate cancer (mHSPC). We retrospectively evaluated data of 70 consecutive patients with mHSPC receiving treatment with either early docetaxel (*n* = 42) or abiraterone acetate (*n* = 28) between July 2018 and April 2021. Skeletal muscle index was calculated from cross-sectional areas of skeletal muscle on baseline computed tomography (CT), defining sarcopenia as a skeletal muscle index of ≤52.4 cm^2^/m^2^. Failure-free survival (FFS), radiographic progression-free survival, and time to prostate-specific antigen (PSA) progression were estimated using the Kaplan–Meier method, and differences in survival probability were compared using the log-rank test. Cox proportional hazards regression analysis was conducted to identify the predictors of clinical outcomes. Patients with sarcopenia (*n* = 47) had shorter FFS than those without sarcopenia (*n* = 23) (median, 20.1 months vs. not reached; log-rank *p* < 0.001). Sarcopenia was independently associated with shorter FFS (hazard ratio (HR), 6.69; 95% confidence interval (CI), 1.57–28.49; *p* = 0.010) and time to PSA progression (HR, 12.91; 95% CI, 1.08–153.85; *p* = 0.043). In conclusion, sarcopenia is an independent prognostic factor for poor FFS and time to PSA progression in patients with mHSPC who receive early docetaxel or abiraterone acetate treatment.

## 1. Introduction

Prostate cancer is the second most common cancer in men and the fifth leading cause of death worldwide [1]. Although androgen deprivation therapy (ADT) has historically been the mainstay of therapy for metastatic hormone-sensitive prostate cancer (mHSPC) showing an initial response, castration-resistant prostate cancer eventually develops within a median of approximately 2–3 years [2]. As metastatic castration-resistant prostate cancer (mCRPC) is associated with a poor prognosis, prolonging the duration of response in mHSPC has been demanded [3]. Since chemohormonal treatment with docetaxel in addition to ADT has been shown to improve overall survival [4,5], several novel hormonal therapies including abiraterone acetate [6], enzalutamide [7], and apalutamide [8] have also been approved, drastically shifting the treatment paradigm in mHSPC.

Sarcopenia, characterized by loss of skeletal muscle quantity or quality, is increasingly recognized as a factor in cancer cachexia syndrome [9]. With several studies reporting its association with a poor prognosis, it has emerged as a potentially modifiable risk factor and an important prognostic predictor in various types of cancers [10,11,12]. The understanding of cancer cachexia also has improved during the past 20 years, as the mechanisms involved in its development continue to be elucidated [13]. However, the mechanism underlying the association between sarcopenia and adverse clinical outcomes in cancer patients remains uncertain, given that the majority of studies on sarcopenia had focused on the clinical relevance without exploring its biological perspective [14]. To provide better treatment strategies in cancer patients, an increased understanding of the underlying mechanism of sarcopenia regarding its impact would be necessary. In this context, recent studies that investigated gene expression status and found its relationship with body composition and clinical outcomes may suggest an interesting way to find a potential link between sarcopenia and its adverse outcomes [15,16].

In prostate cancer, sarcopenia has been reported to be associated with worse survival after radical prostatectomy [17] or radiation therapy [18]. Sarcopenia also seems to be a prognostic factor after systemic treatment in mHSPC [19] and mCRPC [20,21]. However, to the best of our knowledge, its significance has not been determined yet in mHSPC treated with early docetaxel or abiraterone acetate, particularly in association with treatment response. Considering that little is known about prognosticators after the administration of these agents, despite the aforementioned, rapidly evolving management strategies, finding whether sarcopenia has a prognostic value in such patients would be beneficial to help improve the outcomes.

In this study, we hypothesized that sarcopenia could be a significant prognostic factor in patients with mHSPC. Thus, we conducted this study to explore whether sarcopenia determined by cross-sectional imaging has prognostic value in patients with mHSPC receiving treatment with either early docetaxel (chemohormonal treatment) or abiraterone acetate. We also performed RNA sequencing of tumor samples and compared the transcriptomic features of those with and without sarcopenia.

## 2. Materials and Methods

### 2.1. Patients

Our institutional review board (Samsung Medical Center, IRB file No. 2021-05-124) approved this retrospective study and waived the requirement for informed consent. The study was performed in accordance with the Declaration of Helsinki. We reviewed the electronic medical records of 82 consecutive patients who started treatment with early docetaxel or abiraterone acetate between July 2018 and April 2021 for mHSPC. After excluding 12 patients whose height and body weight records were not available, a total of 70 patients (42 treated with docetaxel and 28 with abiraterone acetate) were finally included in the analysis.

In addition to ADT, all patients received one of the following treatments: (1) docetaxel (intravenous infusion over 1 h, 40 mg/m^2^ body surface area, every 2 weeks for up to 12 cycles) with dexamethasone, antiemetics, and prednisone (oral, 5 mg twice daily); (2) abiraterone acetate (oral, 1000 mg as 4 × 250 mg tablets daily) and prednisone (oral, 5 mg once daily). Patients continued to receive treatment until disease progression or unacceptable toxicity. Bone scans and computed tomography (CT) examinations were performed every 8 weeks before and during the first 24 weeks of treatment, and every 12 weeks thereafter.

### 2.2. Image Analysis

**Figure 1:**
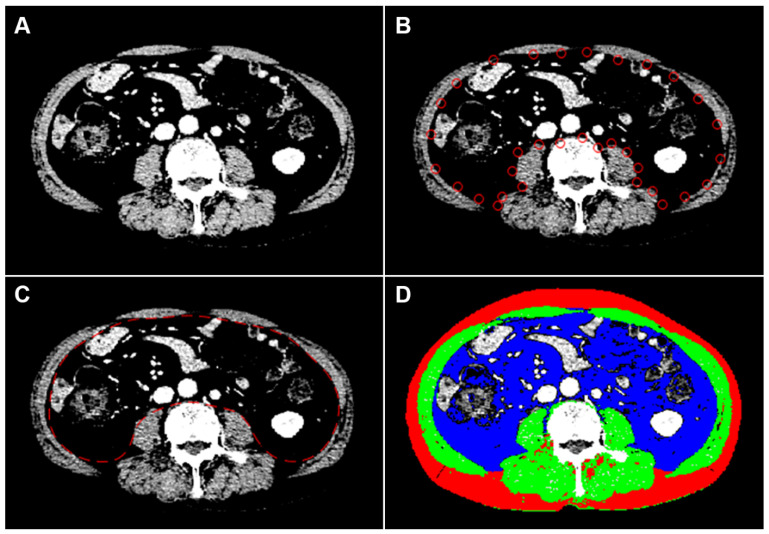
An example of semiautomatic quantification of body composition in a 75-year-old man with metastatic hormone-sensitive prostate cancer. To highlight the muscle boundary, the intensity of the CT image is linearly transformed into 0 to +100 HU (**A**). After semiautomatic manipulation (**B**), the boundary between the muscles and the inner tissues is detected using the active contour method by minimizing a cost function, dividing CT images into inner and outer regions (**C**). Pixels in the fat and muscle are then identified using cut-off values of −300 to −50 HU and −29 to +150 HU, respectively (**D**). The cross-sectional areas of muscle, subcutaneous fat, and visceral fat are measured to be 140.03 cm^2^, 118.69 cm^2^, and 181.37 cm^2^, respectively. Green-colored, red-colored, and blue-colored areas represent muscle, subcutaneous fat, and visceral fat, respectively; CT, computed tomography; HU, Hounsfield unit.

A board-certified radiologist with 7 years of experience in musculoskeletal imaging determined the areas of skeletal muscles, subcutaneous fat, and visceral fat, blinded to patient information. Baseline abdominal CT studies before treatment initiation were analyzed using open-source semi-automated software (BMI_CT, version 1.0; available at https://sourceforge.net/projects/muscle-fat-area-measurement/, accessed on 10 May 2021) based on MATLAB version R2010a (Mathworks Inc., Natick, MA, USA). At the level of the third lumbar vertebrae [22], cross-sectional areas (cm^2^) of skeletal muscles, including the rectus, transverse and oblique abdominal muscles, psoas muscles, paraspinal muscles, subcutaneous fat, and visceral fat, were measured (Figure 1). The patients’ body composition areas (cm^2^) were normalized by dividing by the square of the height (m^2^) of the patient to calculate the skeletal muscle index (SMI) [23], subcutaneous fat index (SFI), and visceral fat index. Visceral-to-subcutaneous fat area ratio was calculated by dividing the area of visceral fat by that of subcutaneous fat. Sarcopenia was defined as an SMI of ≤52.4 cm^2^/m^2^, as proposed by a CT-based sarcopenia study of patients with cancer [24].

### 2.3. Clinical Data Collection

Electrical medical records were reviewed to collect baseline demographics: body weight; height; Gleason score; prostate-specific antigen (PSA) level before and during the treatment; Eastern Cooperative Oncology Group performance status (ECOG PS); clinical stage; and presence of regional lymph node, bone, or visceral metastases.

Patients were classified into low- or high-risk based on the LATITUDE trial, defining high-risk disease as having at least two of the following three criteria: a Gleason score of ≥8, bone lesions ≥3, and the presence of measurable visceral metastasis [6]. They were also stratified according to the CHAARTED criteria, defining visceral metastasis or ≥4 bone lesions with ≥1 beyond the vertebral bodies and pelvis as high-volume, while low-volume included patients that did not meet the above criteria [5].

### 2.4. RNA Sequencing Pre-Process and Gene Set Enrichment Analysis

RNA sequencing data from primary tumor specimens were collected with the patients’ written informed consent as part of another prospective study that was approved by the institutional review board (Samsung Medical Center, IRB file No. 2019-08-012). Prostate needle biopsy specimens at the time of the initial diagnoses were used, irrespective of whether the patients underwent prostatectomy or not, to maintain the consistency of the sample status among the patients. To perform RNA sequencing, total RNA was extracted from 4 µm-thick formalin-fixed, paraffin-embedded sections from each representative primary tumor block containing the highest tumor proportion using an RNA extraction kit, and RNA integrity was verified using a bioanalyzer. The libraries for sequencing were generated with the QuantSeq 3 Library Prep Kit according to the manufacturer’s instructions and sequenced on a HiSeq 2000 system. The reads were mapped to the hg19 human reference genome using STAR with default parameters. The number of reads mapped to each gene was calculated using RNA-Seq by expectation-maximization.

Gene sets related to muscle from the Molecular Signatures Database were used to perform gene set enrichment analysis (GSEA). Single-sample GSEA (ssGSEA) was performed using the “GSVA” package. Data processing and analysis were performed using R/Bioconductor libraries (version 1.4.1106). To confirm the biological roles of differentially expressed genes, we analyzed gene ontology terms using the DAVID website. ssGSEA was performed to characterize the transcriptomic changes using gene sets related to the muscle.

### 2.5. Endpoints

The primary endpoint was failure-free survival (FFS) according to the STAMPEDE trial, defined as the time from treatment initiation to the first of the following events: biochemical failure [4]; progression either locally, in lymph nodes, or distant metastases; or death from prostate cancer. The secondary endpoints were radiographic progression-free survival (PFS) and time to PSA progression based on the Prostate Cancer Working Group 2 criteria [25]. Radiographic PFS was defined as the time from treatment initiation to the first occurrence of either progression by bone scan, CT, or magnetic resonance imaging, as defined by modified RECIST version 1.0. Time to PSA progression was defined as the time from treatment initiation to PSA progression as a 25% increase from the baseline value, along with an increase in the absolute value of 2 ng/mL or more after 12 weeks of treatment.

### 2.6. Statistical Analysis

Patient characteristics were compared between the sarcopenic and non-sarcopenic groups. Continuous variables were compared using the Mann–Whitney test, and categorical variables were compared using the chi-squared test or Fisher’s exact test.

The Kaplan–Meier method with the log-rank test was used to characterize the event–time distributions. A Cox proportional hazards regression model was used to explore whether sarcopenia and other variables were associated with survival outcomes. SFI, visceral fat index, visceral-to-subcutaneous fat area ratio, and baseline PSA level were dichotomized using median values as cutoffs. To find independent predictors, factors with *p* < 0.10 in the univariable tests were entered into the multivariable analyses, with age, baseline PSA level, disease volume, and disease risk being fixed. The interaction term in the Cox proportional hazard regression model was used to determine whether the association between sarcopenia and FFS differed between the docetaxel and abiraterone acetate cohorts.

A t-test was used to evaluate transcriptome changes between samples with and without sarcopenia. All statistical analyses were performed using SPSS Statistics (version 27.0; SPSS Inc., Chicago, IL, USA) and MedCalc^®^ Statistical Software version 20 (MedCalc Software Ltd., Ostend, Belgium). Statistical significance was set at *p* < 0.05.

## 3. Results

The median interval between the CT examination and treatment initiation was 24.5 days (interquartile range (IQR), 17.0–40.0 days). The majority of patients had “de novo” metastasis (already metastatic at the time of diagnosis), only five patients (6.1%) having metachronous metastasis after radical prostatectomy that were performed for curative intent in localized stages. The numbers of patients having ECOG PS of 0, 1, and 2 were 47 (57.3%), 32 (39.0%), and 3 (3.7%), respectively. Bone metastasis was present in 69 patients (84.1%) involving pelvic bones (*n* = 66; 80.5%), vertebrae (*n* = 63; 76.8%), and others (*n* = 58; 70.7%), of which 59 patients (80.0%) had more than 4 metastatic bone lesions. Additionally, 24 (29.3%) patients had visceral metastases in lung (*n* = 19), liver (*n* = 4), pleura (*n* = 3), adrenal gland (*n* = 1), peritoneum (*n* = 1), and ureter (*n* = 1). Among 47 (67.1%) patients, 30 (71.4%) and 17 (60.7%) of docetaxel and abiraterone acetate cohorts, respectively, had sarcopenia, according to the cutoff. The median follow-up duration in all patients was 20.5 months (IQR, 8.9–27.8 months), whereas those in docetaxel and abiraterone acetate cohorts were 25.1 months (IQR, 20.4–30.2 months) and 10.3 months (IQR, 4.9–23.6 months), respectively.

Baseline patient characteristics are shown in Table 1, with a comparison between sarcopenic and non-sarcopenic groups. Compared with the non-sarcopenic group, the sarcopenic group showed significantly lower body mass index, lower prevalence of obesity, lower SMI, lower SFI, lower rate of regional lymph node metastasis, higher rate of bone metastasis, and higher incidence of high-volume and high-risk disease.

### 3.1. Failure-Free Survival

**Figure 2:**
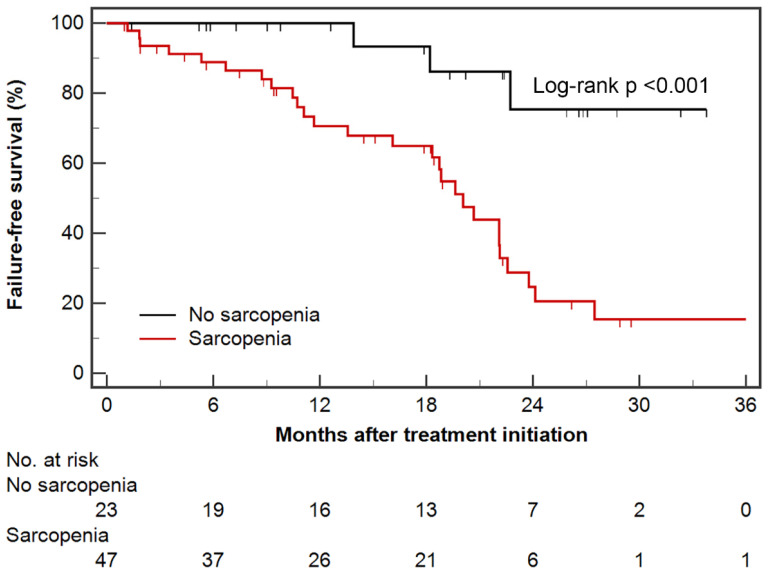
Kaplan–Meier estimates of failure-free survival according to sarcopenic status. Censored data are marked at each line.

In all patients, median FFS was 22.1 months (95% CI, 19.6–27.5 months); the number of events and median FFS in the docetaxel cohort was 24 (57.1%) and 22.1 months (95% CI, 18.8–27.5 months), whereas those in abiraterone acetate cohort were seven (25.0%) and not reached, respectively. Patients with sarcopenia had shorter FFS than those without sarcopenia (median, 20.1 months vs. not reached; log-rank *p* < 0.001) (Figure 2); FFS of sarcopenic groups in both docetaxel (median, 20.7 months vs. not reached; log-rank *p* = 0.041) and abiraterone acetate (median, 11.7 months vs. not reached; log-rank *p* = 0.009) cohorts were shorter than those of non-sarcopenic groups.

In univariable Cox regression analysis, sarcopenia and ECOG PS ≥ 1 were significant prognostic factors for predicting poor FFS, whereas obesity and high SFI (above median) were significantly associated with favorable FFS. Only sarcopenia was an independent prognostic factor associated with shorter FFS on multivariable analysis (Table 2). Although the interaction between sarcopenia and treatment agent showed heterogeneity between docetaxel and abiraterone acetate cohorts (*p* for interaction = 0.016), this significance disappeared after adjusting for follow-up duration (*p* for interaction = 0.130).

### 3.2. Radiographic Progression-Free Survival and Time to PSA Progression

**Figure 3:**
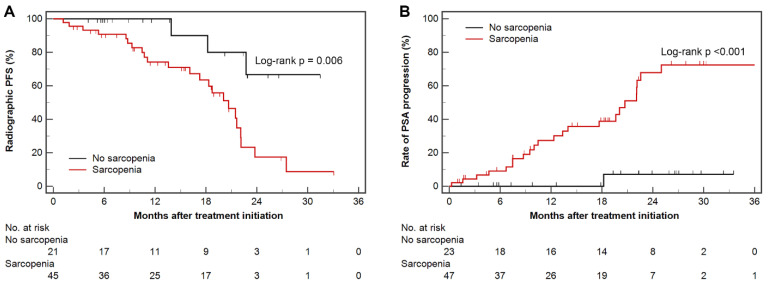
Kaplan–Meier estimates of radiographic progression-free survival (**A**), and rate of PSA progression (**B**) according to sarcopenic status. Censored data are marked at each line. PSA, prostate-specific antigen.

Patients with sarcopenia had shorter radiographic PFS (median, 20.7 months vs. not reached; log-rank *p* = 0.006) and time to PSA progression (median, 20.7 months vs. not reached; log-rank *p* < 0.001) than those without sarcopenia (Figure 3). In univariable Cox regression analysis, sarcopenia and ECOG PS ≥ 1 were significant prognostic factors for predicting poor radiographic PFS; both factors were insignificant in the multivariable analysis (Table 3). For time to PSA progression, sarcopenia, ECOG PS ≥ 1, and high-volume disease were significantly poor prognostic factors, whereas obesity and high SFI (above median) were significantly associated with favorable prognosis. Among them, sarcopenia and ECOG PS ≥ 1 were independently significant (Table 4).

### 3.3. The Characteristics of Transcript between Samples with and without Sarcopenia

**Figure 4:**
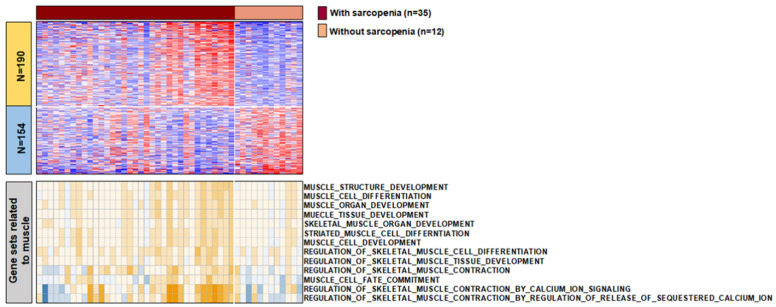
The heatmaps for transcriptomic features of primary tumors according to the presence of sarcopenia. Differentially expressed genes demonstrated between samples with and without sarcopenia (top). Single sample gene set enrichment analysis of 13 muscle-related gene sets in samples with and without sarcopenia (bottom).

To further comprehend the biological role of sarcopenia in mHSPC, we performed RNA sequencing of the primary tumors. Finally, RNA sequencing data of 47 patients were available for specimens obtained through radical prostatectomy (*n* = 5) or prostate needle biopsy (*n* = 42). Among the 35 patients with sarcopenia and 12 patients without sarcopenia, 344 differentially expressed genes were identified (fold difference > 1). Upregulated genes in samples with sarcopenia were associated with morphogenesis of an epithelial sheet, extracellular matrix organization, and extracellular structure organization. Downregulated genes in samples with sarcopenia were associated with metabolic processes (Supplementary Tables S1 and S2). Single-sample gene set enrichment analysis using gene sets related to muscle demonstrated that 13 muscle-related gene sets, including muscle structure development and muscle tissue development, were found to be significantly enriched in samples with sarcopenia (Figure 4).

## 4. Discussion

To our knowledge, this is the first report on the prognostic role of sarcopenia and associated transcriptomic features of primary tumors in patients with mHSPC receiving treatment with either early docetaxel or abiraterone acetate. We analyzed 70 patients with mHSPC and demonstrated that sarcopenia determined by cross-sectional areas of skeletal muscle on baseline CT experienced poor FFS and time to PSA progression, independent of other body composition features and clinical variables. Additionally, RNA sequencing data revealed that the transcriptomic features of tumor samples with and without sarcopenia were significantly different.

Whether sarcopenia is a cause or a consequence of cancer progression remains controversial [26]. Skeletal muscle atrophy in patients experiencing an acute critical illness or cancer [27] raises suspicions for reverse causality. In this study, patients with sarcopenia had a higher prevalence of bone metastasis, high volume, and high-risk disease than those without sarcopenia. Patients with bone metastases would be more vulnerable to deconditioning with more restrictions in their daily activities. In this context, the question of whether the poor prognosis in patients with sarcopenia was attributed to disease burden may persist. However, the fact that most of the patients had ECOG PS of 0 or 1 despite having bone metastasis and high-volume, high-risk disease, and that sarcopenia remained significant after adjusting for other clinical variables might mitigate the effect of reverse causality, although they are not removed. Future investigations using propensity-score matching would aid in identifying this causal relationship. Considering that imaging cannot directly reflect muscle function, which is also essential for the diagnosis of sarcopenia [28], whether sarcopenia determined by nonimaging diagnostic tests are also related to prognosis in patients with mHSPC would be another topic worth investigation.

While both docetaxel [4,5] and abiraterone acetate [6] could be used upfront along with ADT to improve overall survival in patients with mHSPC, no randomized trials have directly compared these agents. In contrast, a direct, randomized, comparative analysis using prospectively collected data from the STAMPETE trials suggested that abiraterone acetate has a PFS advantage over docetaxel, although no significant difference was noted in overall survival [29]. While the adverse impact of sarcopenia may also differ between these two agents, this could not be determined in this study, owing to the relatively short follow-up duration in the abiraterone acetate cohort, which is one of the limitations of this study requiring further validation. In this context, identifying which agent would be beneficial, particularly in patients with sarcopenia, could be an interesting topic for future research.

Interestingly, we observed that obesity and high SFI were associated with favorable FFS and time to PSA progression on univariable analyses. This finding is similar to the unexpected and paradoxical benefit of obesity, termed the “obesity paradox [30]”, that was also documented in mHSPC [31]. Although methodological problems such as reverse causality, collider-stratification or detection bias, other confounding factors, or inadequacy of BMI as an accurate representation of obesity have been considered as possible explanations, the obesity paradox remains poorly understood [32]. As the protective effect of obesity and SFI disappeared after adjusting for other variables including sarcopenia, it could be reasonable to consider that this protective effect is attributed to other confounding factors, of which sarcopenia is possibly the most important. The study by Perna et al. [33], which revealed that increased adiposity is protective regarding muscle loss, also supports this explanation.

Our RNA sequencing results implied that transcriptomic alterations in tumors may be associated with sarcopenia in patients with mHSPC. We observed that gene sets associated with the organization of the extracellular matrix, extracellular structure, and muscle-related gene sets were upregulated and enriched in samples with sarcopenia. Several authors examining muscle samples reported that pathological and adaptive processes in skeletal muscle of patients with sarcopenia are maintained, upregulated, or dysregulated [34,35], similar to our results. However, our result contrasted with previous studies describing that age-dependent muscle catabolic processes and sarcopenia are related to alteration and downregulation of the extracellular matrix [36,37]. In patients with renal cell carcinoma, Ho et al. [38] demonstrated that extracellular matrix genes are upregulated in metastases, implying that its upregulation is a critical molecular event leading to visceral, bone, and soft tissue metastases. Notably, excessive accumulation of tumor extracellular matrix protects the tumor from systemically applied therapeutic agents by impeding the diffusion of the drug into the tumor cells, triggering hypoxia and metabolic stress that promotes pathological signaling to impair drug effectiveness, and driving the epithelial-to-mesenchymal transition [39]. In this regard, our results may indicate that upregulation of genes related to the extracellular matrix plays a pivotal role in adverse clinical outcomes in patients with sarcopenia, partially comparable to a previous study reporting that such patients harbor gene expression associated with more aggressive tumor biology [15].

Moreover, host–tumor interaction could also be a possible explanation, considering that a previous study reported that fatty acid synthase pathway activation in primary tumor is associated with BMI and survival in patients with metastatic renal cell carcinoma [16]. As host inflammatory/immune response is associated with sarcopenia [40,41,42], and skeletal muscle has been known to regulate immunological processes and the inflammatory response, such as T cells, NK cells, and macrophages in human body [43], we believe that body composition changes in the host through sarcopenia might influence tumor biology along with alterations of the transcriptomic network, possibly driven by inflammatory and immunological process. Future studies should address whether tumor transcriptomic features are a potential link between sarcopenia and poor clinical outcomes.

Our study has several limitations. First, it was conducted with a small number of patients, limiting the power of this study. Second, as this study was a retrospective observational study, the causal relationship of sarcopenia could not be determined, as described above. Additionally, residual confounding despite multivariable analyses, or unmeasured confounding factors, could also be possible. Third, the follow-up duration was short, which prohibited the evaluation of overall survival. Fourth, CT attenuation of muscles could not be evaluated, as variable CT protocols were used, although its assessment including Hounsfield unit average calculation has been reported to be associated with patients’ prognosis [44,45,46]. In this context, a subsequent study evaluating both muscle mass and quality would be beneficial. Fifth, selection bias might have influenced our study results, as most of the patients had high-risk and high-volume diseases. Nevertheless, our data highlight the clinical impact of sarcopenia on treatment outcomes in patients with mHSPC treated with either early docetaxel or abiraterone acetate.

## 5. Conclusions

In summary, sarcopenia determined by cross-sectional areas of skeletal muscles on baseline CT is an independent prognostic factor for predicting poor FFS and time to PSA progression in patients with mHSPC receiving treatment with early docetaxel or abiraterone acetate. Transcriptomic differences between primary tumors with and without sarcopenia may have the potential to link sarcopenia and poor clinical outcomes in these patients.

## Figures and Tables

**Table 1:** Baseline characteristics.

Variables	Total (*n* = 70)	No Sarcopenia (*n* = 23)	Sarcopenia (*n* = 47)	*p*
Age (years) *	66.5 (60.0, 73.0)	63.0 (58.0, 70.0)	68.0 (63.3, 73.0)	0.070
BMI (km/m^2^) *	24.3 (21.7, 25.8)	25.6 (25.1, 28.6)	22.4 (21.2, 25.0)	<0.001
Obesity, *n* ^†^	29 (41.4%)	17 (73.9%)	12 (25.5%)	<0.001
SMI (cm^2^/m^2^) *	49.6 (44.9, 53.1)	58.1 (53.8, 62.4)	46.5 (43.7, 48.6)	<0.001
SFI (cm^2^/m^2^) *	38.3 (29.4, 47.9)	48.1 (38.3, 65.3)	34.5 (27.8, 45.5)	<0.001
VFI (cm^2^/m^2^) *	47.2 (30.3, 62.8)	48.5 (37.6, 70.6)	45.3 (25.0, 62.5)	0.165
VSR *	1.09 (0.76, 1.58)	0.93 (0.82, 1.52)	1.16 (0.78, 1.59)	0.516
PSA (ng/mL) *	299.4 (89.6, 801.3)	237.3 (94.4, 540.2)	338.0 (101.7, 1248.7)	0.241
Treatment agent, *n* ^†^				0.353
Docetaxel	42 (60.0%)	12 (52.2%)	30 (63.8%)	
Abiraterone acetate	28 (40.0%)	11 (47.8%)	17 (36.2%)	
ECOG PS > 0, *n* ^†^	34 (48.6%)	10 (43.5%)	24 (51.1%)	0.554
Gleason score ≥ 8, *n* ^‡^	63 (94.0%)	19 (90.5%)	44 (95.7%)	0.584
Stage (cT4), *n* ^†^	37 (55.2%)	16 (69.6%)	21 (47.7%)	0.090
Regional LN metastasis, *n* ^‡^	56 (80.0%)	22 (95.7%)	34 (72.3%)	0.026
Bone metastasis, *n* ^‡^	58 (82.9%)	14 (60.9%)	44 (93.6%)	0.001
Visceral metastasis, *n* ^†^	20 (28.6%)	7 (30.4%)	13 (27.7%)	0.811
High-volume, *n* ^†^	56 (80.0%)	14 (60.9%)	42 (89.4%)	0.006
High-risk, *n* ^‡^	59 (84.3%)	14 (60.9%)	45 (95.7%)	<0.001

BMI, body mass index; SMI, skeletal muscle index; SFI, subcutaneous fat index; VFI, visceral fat index; VSR, visceral-to-subcutaneous fat ratio; PSA, prostate-specific antigen; ECOG PS, Eastern Cooperative Oncology Group performance status; LN, lymph node. * Mann–Whitney test. Numbers are medians and interquartile ranges in curved brackets. ^†^ Chi-squared test. ^‡^ Fischer’s exact test.

**Table 2:** Results of Cox regression analysis for failure-free survival.

Variables (Reference)	Univariable		Multivariable	
	HR (95% CI)	*p*	HR (95% CI)	*p*
Age ≥ 65 years (<65 years)	1.78 (0.82–3.87)	0.145	1.33 (0.55–3.24)	0.529
Obesity (non-obesity)	0.42 (0.19–0.93)	0.033	2.51 (0.60–10.49)	0.207
Sarcopenia (no sarcopenia)	6.18 (1.87–20.44)	0.003	6.69 (1.57–28.49)	0.010
SFI ≥ median (<median)	0.34 (0.16–0.75)	0.007	0.30 (0.08–1.07)	0.063
VFI ≥ median (<median)	1.04 (0.51–2.15)	0.912		
VSR ≥ median (<median)	1.42 (0.69–2.93)	0.342		
PSA ≥ median (<median)	1.17 (0.56–2.41)	0.677	0.91 (0.36–2.30)	0.843
Abiraterone acetate (docetaxel)	0.88 (0.37–2.08)	0.774		
ECOG PS ≥ 1 (0)	2.39 (1.12–5.14)	0.025	2.10 (0.93–4.72)	0.073
Gleason score ≥ 8 (<8)	N/A	0.955		
Stage cT4 (≤cT3)	0.82 (0.38–1.77)	0.618		
Regional LN metastasis (no)	1.47 (0.56–3.85)	0.431		
Bone metastasis (no)	1.27 (0.48–3.31)	0.632		
Visceral metastasis (no)	1.13 (0.51–2.46)	0.768		
High-volume (low-volume)	2.43 (0.84–7.04)	0.102	1.41 (0.24–8.33)	0.704
High-risk (low-risk)	2.95 (0.89–9.78)	0.078	0.911 (0.36–2.30)	0.843

Variables with *p* < 0.10 in the univariable analysis are entered into the multivariable analysis, with age, baseline PSA level, disease volume, and disease risk being fixed.

**Table 3:** Results of Cox regression analysis for radiographic progression-free survival.

Variables (Reference)	Univariable		Multivariable	
	HR (95% CI)	*p*	HR (95% CI)	*p*
Age ≥ 65 years (<65 years)	2.33 (0.98–5.55)	0.055	1.63 (0.63–4.21)	0.314
Obesity (non-obesity)	0.54 (0.23–1.26)	0.155		
Sarcopenia (no sarcopenia)	4.73 (1.40–15.96)	0.012	3.77 (0.95–14.99)	0.060
SFI ≥ median (<median)	0.65 (0.30–1.45)	0.294		
VFI ≥ median (<median)	0.97 (0.44–2.15)	0.944		
VSR ≥ median (<median)	0.90 (0.42–1.96)	0.798		
PSA ≥ median (<median)	1.11 (0.51–2.42)	0.790	0.86 (0.33–2.24)	0.759
Abiraterone acetate (docetaxel)	0.70 (0.28–1.79)	0.463		
ECOG PS ≥ 1 (0)	2.61 (1.12–6.07)	0.026	2.27 (0.96–5.39)	0.063
Gleason score ≥ 8 (<8)	N/A	0.963		
Stage cT4 (≤cT3)	0.96 (0.41–2.22)	0.918		
Regional LN metastasis (no)	0.91 (0.34–2.44)	0.853		
Bone metastasis (no)	1.18 (0.44–3.14)	0.748		
Visceral metastasis (no)	0.97 (0.40–2.33)	0.946		
High-volume (low-volume)	2.15 (0.72–6.41)	0.169	1.17 (0.21–6.53)	0.855
High-risk (low-risk)	2.46 (0.73–8.30)	0.147	0.89 (0.33–2.24)	0.759

Variables with *p* < 0.10 in the univariable analysis are entered into the multivariable analysis, with age, baseline PSA level, disease volume, and disease risk being fixed.

**Table 4:** Results of Cox regression analysis for time to PSA progression.

Variables (Reference)	Univariable		Multivariable	
	HR (95% CI)	*p*	HR (95% CI)	*p*
Age ≥ 65 years (<65 years)	1.95 (0.80–4.75)	0.143	1.41 (0.53–3.74)	0.494
Obesity (non-obesity)	0.34 (0.13–0.87)	0.025	2.53 (0.47–13.47)	0.278
Sarcopenia (no sarcopenia)	16.07 (2.16–119.46)	0.007	12.91 (1.08–153.85)	0.043
SFI ≥ median (<median)	0.31 (0.13–0.75)	0.009	0.29 (0.06–1.34)	0.114
VFI ≥ median (<median)	1.15 (0.51–2.58)	0.734		
VSR ≥ median (<median)	1.75 (0.76–4.01)	0.185		
PSA ≥ median (<median)	1.84 (0.78–4.30)	0.162	1.12 (0.40–3.10)	0.832
Abiraterone acetate (docetaxel)	1.18 (0.48–2.87)	0.720		
ECOG PS ≥ 1 (0)	3.65 (1.44–9.22)	0.006	2.73 (1.04–7.14)	0.041
Gleason score ≥ 8 (<8)	N/A	0.958		
Stage cT4 (≤cT3)	0.59 (0.24–1.43)	0.246		
Regional LN metastasis (no)	2.03 (0.61–6.82)	0.250		
Bone metastasis (no)	6.01 (0.81–44.67)	0.079	0.43 (0.04–5.04)	0.500
Visceral metastasis (no)	1.39 (0.60–3.26)	0.446		
High-volume (low-volume)	8.62 (1.16–64.07)	0.035	1.37 (0.16–11.96)	0.773
High-risk (low-risk)	N/A	0.950	N/A	0.954

Variables with *p* < 0.10 in the univariable analysis are entered into the multivariable analysis, with age, baseline PSA level, disease volume, and disease risk being fixed.

## Data Availability

The data presented in this study are available on request from the corresponding author. The data are not publicly available due to their containing information that could compromise the privacy of research participants.

## Supplementary Materials

The following are available online at https://www.mdpi.com/article/10.3390/cancers13246345/s1, Table S1: Gene ontology using up-regulated genes in sarcopenia, Table S2: Gene ontology using down-regulated genes in sarcopenia.
