# Supplementary material for: Prognostic Impact of Sarcopenia in Patients with Metastatic Hormone-Sensitive Prostate Cancer
Source: Cancers (Basel). 2021 Dec 17;13(24):6345. doi: 10.3390/cancers13246345 (PMC8699789; doi:10.3390/cancers13246345)
Supplement: Supplementary file 1 [file cancers-13-06345-s001.zip › Table S1.pdf]

Table S1. Gene ontology using up-regulated genes in sarcopenia (cutoff: *P* value <0.05)

Category	Term	Count	%	*P* value	Genes
GOTERM_BP_FAT	GO:0002011~morphogenesis of an epithelial sheet	5	2.702703	0.001	*VANGL2, FLRT3, AJUBA, BMP7, CYR61*
GOTERM_BP_FAT	GO:0030198~extracellular matrix organization	11	5.945946	0.001	*HPSE2, ELF3, ITGA3, ITGA2, COL4A6, COL4A5, COL9A3, LAMC2, ITGB6, THSD4, CYR61*
GOTERM_BP_FAT	GO:0043062~extracellular structure organization	11	5.945946	0.002	*HPSE2, ELF3, ITGA3, ITGA2, COL4A6, COL4A5, COL9A3, LAMC2, ITGB6, THSD4, CYR61*
GOTERM_BP_FAT	GO:0007167~enzyme linked receptor protein signaling pathway	20	10.81081	0.002	*KL, IRS1, CAV2, ITGA3, HFE, ARHGEF28, NRG1, BMP7, PRLR, DKK1, CYR61, DNM1, FLRT3, CHN1, COL4A5, SPRY1, ROR1, ATP6V0A4, RNF165, ATP6V1C2*
GOTERM_BP_FAT	GO:0007409~axonogenesis	12	6.486486	0.002	*LRFN5, VANGL2, BCL11A, FLRT3, CHN1, NRXN3, SLITRK6, NTN4, NRG1, BMP7, RNF165, ROBO1*
GOTERM_BP_FAT	GO:0006805~xenobiotic metabolic process	6	3.243243	0.003	*GSTM2, GSTM1, FMO2, CYP3A5, UGT1A6, CES1*
GOTERM_BP_FAT	GO:0048667~cell morphogenesis involved in neuron differentiation	13	7.027027	0.003	*BCL11A, NTN4, NRXN3, NRG1, BMP7, ROBO1, LRFN5, VANGL2, FLRT3, ID2, CHN1, SLITRK6, RNF165*
GOTERM_BP_FAT	GO:0070886~positive regulation of calcineurin-NFAT signaling cascade	3	1.621622	0.003	*AKAP6, NRG1, LMCD1*
GOTERM_BP_FAT	GO:0071466~cellular response to xenobiotic stimulus	6	3.243243	0.003	*GSTM2, GSTM1, FMO2, CYP3A5, UGT1A6, CES1*
GOTERM_BP_FAT	GO:0046578~regulation of Ras protein signal transduction	8	4.324324	0.004	*RASA4B, ITGA3, RASA4, ARHGEF28, ARHGEF4, SPRY1, NRG1, ROBO1*
GOTERM_BP_FAT	GO:0007265~Ras protein signal transduction	10	5.405405	0.004	*VANGL2, RASA4B, ITGA3, RASA4, PLK2, ARHGEF28, ARHGEF4, SPRY1, NRG1, ROBO1*
GOTERM_BP_FAT	GO:0061564~axon development	12	6.486486	0.004	*LRFN5, VANGL2, BCL11A, FLRT3, CHN1, NRXN3, SLITRK6, NTN4, NRG1, BMP7, RNF165, ROBO1*
GOTERM_BP_FAT	GO:0009410~response to xenobiotic stimulus	6	3.243243	0.004	*GSTM2, GSTM1, FMO2, CYP3A5, UGT1A6, CES1*
GOTERM_BP_FAT	GO:0017144~drug metabolic process	4	2.162162	0.005	*CYP4B1, FMO2, CYP4F12, CYP3A5*
GOTERM_BP_FAT	GO:0007264~small GTPase mediated signal transduction	13	7.027027	0.005	*ARL10, RASA4B, ITGA3, PLK2, ARHGEF28, NRG1, RND3, ROBO1, VANGL2, RASA4, ARHGEF4, RAB38, SPRY1*
GOTERM_BP_FAT	GO:0031581~hemidesmosome assembly	3	1.621622	0.006	*DST, LAMC2, KRT5*
GOTERM_BP_FAT	GO:0007411~axon guidance	8	4.324324	0.006	*VANGL2, FLRT3, CHN1, NRXN3, NTN4, BMP7, RNF165, ROBO1*
GOTERM_BP_FAT	GO:0097485~neuron projection guidance	8	4.324324	0.006	*VANGL2, FLRT3, CHN1, NRXN3, NTN4, BMP7, RNF165, ROBO1*
GOTERM_BP_FAT	GO:0007369~gastrulation	7	3.783784	0.006	*OSR2, VANGL2, DUSP1, ITGA3, ITGA2, BMP7, DKK1*
GOTERM_BP_FAT	GO:0060429~epithelium development	20	10.81081	0.006	*KRT4, ITGA2, NTN4, NRG1, BMP7, PRLR, DKK1, CYR61, ROBO1, VANGL2, ELF3, FLRT3, KRT16, ID2, SLITRK6, SPRY1, ROR1, AJUBA, CES1, KRT6A*
GOTERM_BP_FAT	GO:0050850~positive regulation of calcium-mediated signaling	4	2.162162	0.007	*GSTM2, AKAP6, NRG1, LMCD1*
GOTERM_BP_FAT	GO:0050808~synapse organization	8	4.324324	0.007	*NLGN4Y, CACNB2, FLRT3, NRXN3, SLITRK6, COL4A5, NRG1, DKK1*
GOTERM_BP_FAT	GO:0048729~tissue morphogenesis	14	7.567568	0.007	*ITGA3, ITGA2, NTN4, NRG1, BMP7, DKK1, CYR61, VANGL2, FLRT3, KRT16, SPRY1, ROR1, AJUBA, KRT6A*
GOTERM_BP_FAT	GO:0046580~negative regulation of Ras protein signal transduction	4	2.162162	0.007	*RASA4B, ITGA3, RASA4, SPRY1*
GOTERM_BP_FAT	GO:0042330~taxis	13	7.027027	0.007	*ITGA2, NTN4, NRXN3, NRG1, BMP7, CYR61, ROBO1, VANGL2, FLRT3, ID2, CHN1, S100A14, RNF165*
GOTERM_BP_FAT	GO:0051058~negative regulation of small GTPase mediated signal transduction	4	2.162162	0.008	*RASA4B, ITGA3, RASA4, SPRY1*
GOTERM_BP_FAT	GO:0051056~regulation of small GTPase mediated signal transduction	9	4.864865	0.008	*RASA4B, ITGA3, RASA4, ARHGEF28, CHN1, ARHGEF4, SPRY1, NRG1, ROBO1*
GOTERM_BP_FAT	GO:0008544~epidermis development	9	4.864865	0.009	*VANGL2, ELF3, KRT16, SLITRK6, LAMC2, KRT5, DKK1, EDA2R, ZNF750*
GOTERM_BP_FAT	GO:0030879~mammary gland development	6	3.243243	0.009	*ELF3, ID2, ITGA2, NRG1, PRLR, ROBO1*
GOTERM_BP_FAT	GO:0048598~embryonic morphogenesis	13	7.027027	0.009	*OSR2, DUSP1, ITGA3, ITGA2, FOXI1, BMP7, DKK1, CYR61, VANGL2, FLRT3, ID2, SLITRK6, SP8*
GOTERM_BP_FAT	GO:0007169~transmembrane receptor protein tyrosine kinase signaling pathway	14	7.567568	0.010	*KL, IRS1, CAV2, ARHGEF28, NRG1, PRLR, DNM1, FLRT3, CHN1, COL4A5, SPRY1, ROR1, ATP6V0A4, ATP6V1C2*
GOTERM_BP_FAT	GO:0071495~cellular response to endogenous stimulus	21	11.35135	0.010	*KL, GSTM2, DUSP1, IRS1, CAV2, ITGA3, HFE, LMO3, AKAP6, RORB, BMP7, PRLR, DKK1, CYR61, CPEB1, FLRT3, COL4A6, SPRY1, ATP6V0A4, RNF165, ATP6V1C2*
GOTERM_BP_FAT	GO:0007044~cell-substrate junction assembly	5	2.702703	0.010	*DST, ITGA2, LAMC2, KRT5, AJUBA*
GOTERM_BP_FAT	GO:0009790~embryo development	18	9.72973	0.010	*OSR2, DUSP1, ITGA3, NRK, ITGA2, FOXI1, NRG1, BMP7, DKK1, CYR61, STOX2, KRT19, VANGL2, ELF3, FLRT3, ID2, SLITRK6, SP8*
GOTERM_BP_FAT	GO:0070887~cellular response to chemical stimulus	38	20.54054	0.011	*IRS1, HFE, AKAP6, RORB, CYP3A5, CYR61, EDA2R, ROBO1, DUOX1, FLRT3, ATP6V0A4, S100A14, PRODH, ATP6V1C2, UGT1A6, KL, GSTM2, GSTM1, CAV2, ITGA3, DUSP1, LMO3, ITGA2, KRT13, TNFRSF10C, FMO2, BMP7, DKK1, PRLR, IL17RB, CPEB1, CYP24A1, RASA4, ID2, COL4A6, SPRY1, RNF165, CES1*
GOTERM_BP_FAT	GO:0070884~regulation of calcineurin-NFAT signaling cascade	3	1.621622	0.011	*AKAP6, NRG1, LMCD1*
GOTERM_BP_FAT	GO:0051962~positive regulation of nervous system development	11	5.945946	0.012	*BCL11A, FLRT3, ITGA3, ID2, DUOXA1, NRXN3, SLITRK6, NRG1, BMP7, DKK1, ROBO1*
GOTERM_BP_FAT	GO:1902532~negative regulation of intracellular signal transduction	11	5.945946	0.012	*GSTM2, RASA4B, DUSP1, FLRT3, ITGA3, RASA4, LMO3, WWC2, SPRY1, AJUBA, BMP7*
GOTERM_BP_FAT	GO:1902531~regulation of intracellular signal transduction	27	14.59459	0.013	*IRS1, ARHGEF28, NRK, WWC2, AKAP6, CYR61, EDA2R, ROBO1, FLRT3, CHN1, LMCD1, KL, GSTM2, RASA4B, CAV2, ITGA3, DUSP1, LMO3, PLK2, NRG1, BMP7, PRLR, VANGL2, RASA4, ARHGEF4, SPRY1, AJUBA*
GOTERM_BP_FAT	GO:0042537~benzene-containing compound metabolic process	3	1.621622	0.016	*GSTM2, GSTM1, CYP4B1*
GOTERM_BP_FAT	GO:0048812~neuron projection morphogenesis	12	6.486486	0.016	*LRFN5, VANGL2, BCL11A, FLRT3, CHN1, NRXN3, SLITRK6, NTN4, NRG1, BMP7, RNF165, ROBO1*
GOTERM_BP_FAT	GO:0051960~regulation of nervous system development	15	8.108108	0.016	*BCL11A, ITGA3, PLK2, DIXDC1, NRXN3, NRG1, BMP7, DKK1, ROBO1, VANGL2, FLRT3, ID2, DUOXA1, CHN1, SLITRK6*
GOTERM_BP_FAT	GO:0090092~regulation of transmembrane receptor protein serine/threonine kinase signaling pathway	7	3.783784	0.017	*CAV2, ITGA3, HFE, BMP7, DKK1, CYR61, RNF165*
GOTERM_BP_FAT	GO:0033173~calcineurin-NFAT signaling cascade	3	1.621622	0.017	*AKAP6, NRG1, LMCD1*
GOTERM_BP_FAT	GO:0009968~negative regulation of signal transduction	19	10.27027	0.017	*GSTM2, RASA4B, DUSP1, IRS1, CAV2, ITGA3, LMO3, WWC2, DDIT4L, NRG1, KIF7, BMP7, DKK1, DNM1, ROBO1, RASA4, FLRT3, SPRY1, AJUBA*
GOTERM_BP_FAT	GO:0006935~chemotaxis	12	6.486486	0.018	*VANGL2, FLRT3, ITGA2, CHN1, NRXN3, NTN4, NRG1, S100A14, BMP7, CYR61, RNF165, ROBO1*
GOTERM_BP_FAT	GO:0009967~positive regulation of signal transduction	23	12.43243	0.018	*KL, GSTM2, IRS1, CAV2, HFE, LMO3, PLK2, NRK, DIXDC1, AKAP6, NRG1, KIF7, BMP7, PRLR, CYR61, EDA2R, ROBO1, VANGL2, CHN1, AJUBA, LMCD1, RNF165, ATP6V1C2*
GOTERM_BP_FAT	GO:1901687~glutathione derivative biosynthetic process	3	1.621622	0.019	*GSTM2, GSTM1, GSTM5*
GOTERM_BP_FAT	GO:1901685~glutathione derivative metabolic process	3	1.621622	0.019	*GSTM2, GSTM1, GSTM5*
GOTERM_BP_FAT	GO:0010648~negative regulation of cell communication	20	10.81081	0.019	*GSTM2, RASA4B, DUSP1, IRS1, CAV2, ITGA3, LMO3, PLK2, WWC2, DDIT4L, NRG1, KIF7, BMP7, DKK1, DNM1, ROBO1, RASA4, FLRT3, SPRY1, AJUBA*

GOTERM_BP_FAT	GO:0023057~negative regulation of signaling	20	10.81081	0.019	*GSTM2, RASA4B, DUSP1, IRS1, CAV2, ITGA3, LMO3, PLK2, WWC2, DDIT4L, NRG1, KIF7, BMP7, DKK1, DNM1, ROBO1, RASA4, FLRT3, SPRY1, AJUBA*
GOTERM_BP_FAT	GO:0010243~response to organonitrogen compound	15	8.108108	0.020	*KL, GSTM2, DUSP1, IRS1, CAV2, ITGA2, AKAP6, BMP7, PRLR, DUOX1, CPEB1, PPP1R1B, COL4A6, ATP6V0A4, ATP6V1C2*
GOTERM_BP_FAT	GO:0060411~cardiac septum morphogenesis	4	2.162162	0.023	*VANGL2, ID2, BMP7, CYR61*
GOTERM_BP_FAT	GO:0009719~response to endogenous stimulus	24	12.97297	0.023	*KL, GSTM2, DUSP1, IRS1, CAV2, ITGA3, HFE, LMO3, ITGA2, AKAP6, RORB, BMP7, PRLR, DKK1, CYR61, DUOX1, CPEB1, FLRT3, PPP1R1B, COL4A6, SPRY1, ATP6V0A4, RNF165, ATP6V1C2*
GOTERM_BP_FAT	GO:0008286~insulin receptor signaling pathway	5	2.702703	0.024	*KL, IRS1, CAV2, ATP6V0A4, ATP6V1C2*
GOTERM_BP_FAT	GO:0010647~positive regulation of cell communication	24	12.97297	0.025	*KL, GSTM2, IRS1, CAV2, HFE, LMO3, PLK2, NRK, ITGA2, DIXDC1, AKAP6, NRG1, KIF7, BMP7, PRLR, CYR61, EDA2R, ROBO1, VANGL2, CHN1, AJUBA, LMCD1, RNF165, ATP6V1C2*
GOTERM_BP_FAT	GO:1901698~response to nitrogen compound	16	8.648649	0.026	*KL, GSTM2, GSTM1, DUSP1, IRS1, CAV2, ITGA2, AKAP6, BMP7, PRLR, DUOX1, CPEB1, PPP1R1B, COL4A6, ATP6V0A4, ATP6V1C2*
GOTERM_BP_FAT	GO:2000725~regulation of cardiac muscle cell differentiation	3	1.621622	0.026	*AKAP6, NRG1, DKK1*
GOTERM_BP_FAT	GO:0023056~positive regulation of signaling	24	12.97297	0.026	*KL, GSTM2, IRS1, CAV2, HFE, LMO3, PLK2, NRK, ITGA2, DIXDC1, AKAP6, NRG1, KIF7, BMP7, PRLR, CYR61, EDA2R, ROBO1, VANGL2, CHN1, AJUBA, LMCD1, RNF165, ATP6V1C2*
GOTERM_BP_FAT	GO:0007507~heart development	11	5.945946	0.026	*FHOD3, VANGL2, FLRT3, ITGA3, ID2, AKAP6, NRG1, BMP7, DKK1, CYR61, ROBO1*
GOTERM_BP_FAT	GO:0001704~formation of primary germ layer	5	2.702703	0.027	*DUSP1, ITGA3, ITGA2, BMP7, DKK1*
GOTERM_BP_FAT	GO:0003206~cardiac chamber morphogenesis	5	2.702703	0.027	*VANGL2, ID2, NRG1, BMP7, CYR61*
GOTERM_BP_FAT	GO:0045995~regulation of embryonic development	5	2.702703	0.027	*OSR2, VANGL2, NRK, BMP7, DKK1*
GOTERM_BP_FAT	GO:0090383~phagosome acidification	3	1.621622	0.028	*RAB38, ATP6V0A4, ATP6V1C2*
GOTERM_BP_FAT	GO:0070458~cellular detoxification of nitrogen compound	2	1.081081	0.029	*GSTM2, GSTM1*
GOTERM_BP_FAT	GO:0001707~mesoderm formation	4	2.162162	0.029	*ITGA3, ITGA2, BMP7, DKK1*
GOTERM_BP_FAT	GO:0000904~cell morphogenesis involved in differentiation	14	7.567568	0.030	*BCL11A, NTN4, NRXN3, NRG1, BMP7, DKK1, ROBO1, LRFN5, VANGL2, FLRT3, ID2, CHN1, SLITRK6, RNF165*
GOTERM_BP_FAT	GO:0048332~mesoderm morphogenesis	4	2.162162	0.031	*ITGA3, ITGA2, BMP7, DKK1*
GOTERM_BP_FAT	GO:0030278~regulation of ossification	6	3.243243	0.031	*KL, OSR2, ID2, RORB, BMP7, CYR61*
GOTERM_BP_FAT	GO:0048738~cardiac muscle tissue development	6	3.243243	0.034	*FHOD3, ID2, AKAP6, NRG1, BMP7, DKK1*
GOTERM_BP_FAT	GO:0090596~sensory organ morphogenesis	7	3.783784	0.034	*OSR2, VANGL2, KRT13, FOXI1, SLITRK6, RORB, BMP7*
GOTERM_BP_FAT	GO:0032869~cellular response to insulin stimulus	6	3.243243	0.035	*KL, CPEB1, IRS1, CAV2, ATP6V0A4, ATP6V1C2*
GOTERM_BP_FAT	GO:1901021~positive regulation of calcium ion transmembrane transporter activity	3	1.621622	0.036	*CACNB2, GSTM2, AKAP6*
GOTERM_BP_FAT	GO:0050890~cognition	7	3.783784	0.036	*NLGN4Y, ITGA3, PLK2, PPP1R1B, NRXN3, TMPRSS11E, DKK1*
GOTERM_BP_FAT	GO:0007416~synapse assembly	5	2.702703	0.037	*NLGN4Y, FLRT3, NRXN3, SLITRK6, NRG1*
GOTERM_BP_FAT	GO:0044708~single-organism behavior	9	4.864865	0.037	*NLGN4Y, ITGA3, PLK2, ID2, PPP1R1B, NRXN3, SLITRK6, DKK1, DNM1*
GOTERM_BP_FAT	GO:0048333~mesodermal cell differentiation	3	1.621622	0.038	*ITGA3, ITGA2, DKK1*
GOTERM_BP_FAT	GO:0048016~inositol phosphate-mediated signaling	3	1.621622	0.038	*AKAP6, NRG1, LMCD1*
GOTERM_BP_FAT	GO:0018916~nitrobenzene metabolic process	2	1.081081	0.038	*GSTM2, GSTM1*
GOTERM_BP_FAT	GO:1900138~negative regulation of phospholipase A2 activity	2	1.081081	0.038	*ANXA8L1, ANXA8*
GOTERM_BP_FAT	GO:0050848~regulation of calcium-mediated signaling	4	2.162162	0.039	*GSTM2, AKAP6, NRG1, LMCD1*
GOTERM_BP_FAT	GO:0050877~neurological system process	20	10.81081	0.040	*NLGN4Y, PRRT2, PDE1C, ITGA3, PLK2, ITGA2, NRXN3, OR5P3, NRG1, OR5P2, RORB, TMPRSS11E, DKK1, DNM1, SCNN1G, CACNB2, PPP1R1B, SLITRK6, ATP6V0A4, TRPM3*
GOTERM_BP_FAT	GO:0071417~cellular response to organonitrogen compound	10	5.405405	0.041	*KL, GSTM2, CPEB1, IRS1, CAV2, COL4A6, AKAP6, ATP6V0A4, PRLR, ATP6V1C2*
GOTERM_BP_FAT	GO:0003156~regulation of organ formation	3	1.621622	0.042	*SPRY1, BMP7, DKK1*
GOTERM_BP_FAT	GO:0044255~cellular lipid metabolic process	17	9.189189	0.043	*KL, GSTM2, ST6GALNAC2, INPP1, IRS1, ACOT11, NRG1, CYP3A5, CYP4F12, CYR61, ACSF2, ST3GAL5, SPTSSB, BCO2, AJUBA, CES1, UGT1A6*
GOTERM_BP_FAT	GO:0030509~BMP signaling pathway	5	2.702703	0.043	*HFE, BMP7, DKK1, CYR61, RNF165*
GOTERM_BP_FAT	GO:0045664~regulation of neuron differentiation	11	5.945946	0.044	*BCL11A, ITGA3, PLK2, ID2, DUOXA1, CHN1, DIXDC1, NRG1, BMP7, DKK1, ROBO1*
GOTERM_BP_FAT	GO:0030510~regulation of BMP signaling pathway	4	2.162162	0.044	*ITGA3, DKK1, CYR61, RNF165*
GOTERM_BP_FAT	GO:0048666~neuron development	16	8.648649	0.045	*BCL11A, ITGA3, PLK2, NTN4, NRXN3, NRG1, RORB, BMP7, ROBO1, LRFN5, VANGL2, FLRT3, ID2, CHN1, SLITRK6, RNF165*
GOTERM_BP_FAT	GO:1905207~regulation of cardiocyte differentiation	3	1.621622	0.047	*AKAP6, NRG1, DKK1*
GOTERM_BP_FAT	GO:0051410~detoxification of nitrogen compound	2	1.081081	0.047	*GSTM2, GSTM1*
GOTERM_BP_FAT	GO:0044272~sulfur compound biosynthetic process	6	3.243243	0.047	*GSTM2, GSTM1, VANGL2, ACOT11, ACSF2, GSTM5*
GOTERM_BP_FAT	GO:0030182~neuron differentiation	19	10.27027	0.047	*BCL11A, ITGA3, PLK2, NTN4, DIXDC1, NRXN3, NRG1, RORB, BMP7, DKK1, ROBO1, LRFN5, VANGL2, FLRT3, ID2, DUOXA1, CHN1, SLITRK6, RNF165*
GOTERM_BP_FAT	GO:0007166~cell surface receptor signaling pathway	35	18.91892	0.048	*IRS1, HFE, ARHGEF28, DIXDC1, CYR61, EDA2R, ROBO1, DUOX1, FLRT3, CHN1, ATP6V0A4, ITGB6, GRIA3, TRPM3, ATP6V1C2, EVC2, KL, DST, CAV2, ITGA3, ITGA2, TNFRSF10C, NRG1, KIF7, BMP7, DKK1, PRLR, DNM1, IL17RB, KRT19, VANGL2, COL4A5, SPRY1, ROR1, RNF165*
GOTERM_BP_FAT	GO:0048568~embryonic organ development	9	4.864865	0.048	*OSR2, KRT19, VANGL2, NRK, ID2, FOXI1, SLITRK6, BMP7, CYR61*
GOTERM_BP_FAT	GO:0045778~positive regulation of ossification	4	2.162162	0.049	*KL, OSR2, BMP7, CYR61*
GOTERM_BP_FAT	GO:0030501~positive regulation of bone mineralization	3	1.621622	0.049	*KL, OSR2, BMP7*
GOTERM_BP_FAT	GO:0055067~monovalent inorganic cation homeostasis	5	2.702703	0.049	*SCNN1G, RAB38, ATP6V0A4, CYP4F12, ATP6V1C2*
GOTERM_BP_FAT	GO:0048584~positive regulation of response to stimulus	28	15.13514	0.049	*IRS1, HFE, NRK, DIXDC1, AKAP6, CYR61, EDA2R, ROBO1, CHN1, S100A14, LMCD1, ATP6V1C2, KL, GSTM2, SCARA3, CAV2, LMO3, PLK2, ITGA2, NRG1, KIF7, BMP7, PRLR, IL17RB, VANGL2, POLR3G, AJUBA, RNF165*
