# Supplementary material for: Prognostic Impact of Sarcopenia in Patients with Metastatic Hormone-Sensitive Prostate Cancer
Source: Cancers (Basel). 2021 Dec 17;13(24):6345. doi: 10.3390/cancers13246345 (PMC8699789; doi:10.3390/cancers13246345)
Supplement: Supplementary file 1 [file cancers-13-06345-s001.zip › Table S2.pdf]

**Table S2. Gene ontology using down-regulated genes in sarcopenia (cutoff: *P* value <0.05)**

| Category | Term | Count | % | *P* value | Genes |
|---|---|---|---|---|---|
| GOTERM_BP_FAT | GO:0019752~carboxylic acid metabolic process | 18 | 13.8462 | <0.001 | *CYP2J2, LYPLA1, XBP1, PLA2G4F, ELOVL5, DCXR, SORD, MIF, ACAT2, GSTZ1, ALDH1A3, PSAT1, EHHADH, PCCB, SMS, CRYL1, GNE, SNCA* |
| GOTERM_BP_FAT | GO:0043436~oxoacid metabolic process | 18 | 13.8462 | <0.001 | *CYP2J2, LYPLA1, XBP1, PLA2G4F, ELOVL5, DCXR, SORD, MIF, ACAT2, GSTZ1, ALDH1A3, PSAT1, EHHADH, PCCB, SMS, CRYL1, GNE, SNCA* |
| GOTERM_BP_FAT | GO:0032787~monocarboxylic acid metabolic process | 14 | 10.7692 | <0.001 | *CYP2J2, LYPLA1, XBP1, PLA2G4F, ELOVL5, DCXR, SORD, MIF, ACAT2, ALDH1A3, EHHADH, PCCB, CRYL1, SNCA* |
| GOTERM_BP_FAT | GO:0006082~organic acid metabolic process | 18 | 13.8462 | <0.001 | *CYP2J2, LYPLA1, XBP1, PLA2G4F, ELOVL5, DCXR, SORD, MIF, ACAT2, GSTZ1, ALDH1A3, PSAT1, EHHADH, PCCB, SMS, CRYL1, GNE, SNCA* |
| GOTERM_BP_FAT | GO:0060337~type I interferon signaling pathway | 6 | 4.61538 | <0.001 | *STAT1, OAS2, OAS3, IFI6, ISG15, IRF9* |
| GOTERM_BP_FAT | GO:0071357~cellular response to type I interferon | 6 | 4.61538 | <0.001 | *STAT1, OAS2, OAS3, IFI6, ISG15, IRF9* |
| GOTERM_BP_FAT | GO:0006631~fatty acid metabolic process | 11 | 8.46154 | <0.001 | *CYP2J2, LYPLA1, XBP1, PLA2G4F, ELOVL5, EHHADH, PCCB, CRYL1, MIF, ACAT2, SNCA* |
| GOTERM_BP_FAT | GO:0034340~response to type I interferon | 6 | 4.61538 | <0.001 | *STAT1, OAS2, OAS3, IFI6, ISG15, IRF9* |
| GOTERM_BP_FAT | GO:0044283~small molecule biosynthetic process | 12 | 9.23077 | <0.001 | *SEC14L2, ALDH1A3, XBP1, PLA2G4F, PSAT1, ELOVL5, RFK, PCCB, SORD, DHCR7, MIF, SNCA* |
| GOTERM_BP_FAT | GO:0051167~xylulose 5-phosphate metabolic process | 3 | 2.30769 | <0.001 | *DCXR, SORD, CRYL1* |
| GOTERM_BP_FAT | GO:1901159~xylulose 5-phosphate biosynthetic process | 3 | 2.30769 | <0.001 | *DCXR, SORD, CRYL1* |
| GOTERM_BP_FAT | GO:0019640~glucuronate catabolic process to xylulose 5-phosphate | 3 | 2.30769 | <0.001 | *DCXR, SORD, CRYL1* |
| GOTERM_BP_FAT | GO:0006064~glucuronate catabolic process | 3 | 2.30769 | <0.001 | *DCXR, SORD, CRYL1* |
| GOTERM_BP_FAT | GO:0072329~monocarboxylic acid catabolic process | 6 | 4.61538 | 0.001 | *EHHADH, DCXR, PCCB, SORD, CRYL1, ACAT2* |
| GOTERM_BP_FAT | GO:0036498~IRE1-mediated unfolded protein response | 5 | 3.84615 | 0.001 | *XBP1, SERP1, DNAJB9, WIPI1, PDIA5* |
| GOTERM_BP_FAT | GO:0046395~carboxylic acid catabolic process | 7 | 5.38462 | 0.002 | *GSTZ1, EHHADH, DCXR, PCCB, SORD, CRYL1, ACAT2* |
| GOTERM_BP_FAT | GO:0030968~endoplasmic reticulum unfolded protein response | 6 | 4.61538 | 0.002 | *XBP1, SERP1, DNAJB9, WIPI1, PDIA5, HSP90B1* |
| GOTERM_BP_FAT | GO:0034620~cellular response to unfolded protein | 6 | 4.61538 | 0.002 | *XBP1, SERP1, DNAJB9, WIPI1, PDIA5, HSP90B1* |
| GOTERM_BP_FAT | GO:0006629~lipid metabolic process | 19 | 14.6154 | 0.002 | *CYP2J2, LYPLA1, XBP1, PLA2G4F, ELOVL5, MBOAT2, MIF, ACAT2, SULT2B1, SEC14L2, INPP4B, ALDH1A3, RDH11, EHHADH, PCCB, CRYL1, DHCR7, MPDU1, SNCA* |
| GOTERM_BP_FAT | GO:0044255~cellular lipid metabolic process | 16 | 12.3077 | 0.003 | *CYP2J2, LYPLA1, XBP1, PLA2G4F, ELOVL5, MBOAT2, MIF, ACAT2, INPP4B, ALDH1A3, RDH11, EHHADH, PCCB, CRYL1, MPDU1, SNCA* |
| GOTERM_BP_FAT | GO:0044712~single-organism catabolic process | 14 | 10.7692 | 0.003 | *XBP1, PLA2G4F, DCXR, SORD, ACAT2, HSP90B1, GSTZ1, EHHADH, PCCB, CRYL1, DNAJB9, ADPRM, CTBS, PDE9A* |
| GOTERM_BP_FAT | GO:0035967~cellular response to topologically incorrect protein | 6 | 4.61538 | 0.003 | *XBP1, SERP1, DNAJB9, WIPI1, PDIA5, HSP90B1* |
| GOTERM_BP_FAT | GO:0016054~organic acid catabolic process | 7 | 5.38462 | 0.003 | *GSTZ1, EHHADH, DCXR, PCCB, SORD, CRYL1, ACAT2* |
| GOTERM_BP_FAT | GO:0030003~cellular cation homeostasis | 11 | 8.46154 | 0.004 | *MCUR1, SCO1, CHP1, TNNI3, TRPM8, ATP6V0E2, GPR89B, SLC39A4, HSP90B1, TRPM4, SNCA* |
| GOTERM_BP_FAT | GO:0006873~cellular ion homeostasis | 11 | 8.46154 | 0.005 | *MCUR1, SCO1, CHP1, TNNI3, TRPM8, ATP6V0E2, GPR89B, SLC39A4, HSP90B1, TRPM4, SNCA* |
| GOTERM_BP_FAT | GO:0006986~response to unfolded protein | 6 | 4.61538 | 0.005 | *XBP1, SERP1, DNAJB9, WIPI1, PDIA5, HSP90B1* |
| GOTERM_BP_FAT | GO:0050801~ion homeostasis | 12 | 9.23077 | 0.005 | *MCUR1, SCO1, IFI6, CHP1, TNNI3, TRPM8, ATP6V0E2, GPR89B, SLC39A4, HSP90B1, TRPM4, SNCA* |
| GOTERM_BP_FAT | GO:0055082~cellular chemical homeostasis | 12 | 9.23077 | 0.006 | *XBP1, MCUR1, SCO1, CHP1, TNNI3, TRPM8, ATP6V0E2, GPR89B, SLC39A4, HSP90B1, TRPM4, SNCA* |
| GOTERM_BP_FAT | GO:0044282~small molecule catabolic process | 8 | 6.15385 | 0.006 | *GSTZ1, EHHADH, DCXR, PCCB, SORD, CRYL1, ADPRM, ACAT2* |
| GOTERM_BP_FAT | GO:0044248~cellular catabolic process | 20 | 15.3846 | 0.007 | *XBP1, PLA2G4F, DCXR, SORD, ISG15, ACAT2, HSP90B1, GSTZ1, HERC3, SCO1, OAS2, ANPEP, EHHADH, PCCB, CRYL1, DNAJB9, ADPRM, PDE9A, RNF187, SNCA* |
| GOTERM_BP_FAT | GO:0046394~carboxylic acid biosynthetic process | 7 | 5.38462 | 0.007 | *ALDH1A3, XBP1, PLA2G4F, PSAT1, ELOVL5, PCCB, MIF* |
| GOTERM_BP_FAT | GO:0072330~monocarboxylic acid biosynthetic process | 6 | 4.61538 | 0.007 | *ALDH1A3, XBP1, PLA2G4F, ELOVL5, PCCB, MIF* |
| GOTERM_BP_FAT | GO:0019725~cellular homeostasis | 13 | 10 | 0.007 | *XBP1, MCUR1, GPR89B, PDIA5, HSP90B1, SCO1, CHP1, TNNI3, TRPM8, ATP6V0E2, SLC39A4, TRPM4, SNCA* |
| GOTERM_BP_FAT | GO:0035966~response to topologically incorrect protein | 6 | 4.61538 | 0.007 | *XBP1, SERP1, DNAJB9, WIPI1, PDIA5, HSP90B1* |
| GOTERM_BP_FAT | GO:0055080~cation homeostasis | 11 | 8.46154 | 0.008 | *MCUR1, SCO1, CHP1, TNNI3, TRPM8, ATP6V0E2, GPR89B, SLC39A4, HSP90B1, TRPM4, SNCA* |
| GOTERM_BP_FAT | GO:0016053~organic acid biosynthetic process | 7 | 5.38462 | 0.009 | *ALDH1A3, XBP1, PLA2G4F, PSAT1, ELOVL5, PCCB, MIF* |
| GOTERM_BP_FAT | GO:0006066~alcohol metabolic process | 7 | 5.38462 | 0.010 | *SEC14L2, INPP4B, ALDH1A3, RDH11, SORD, DHCR7, SNCA* |
| GOTERM_BP_FAT | GO:0098771~inorganic ion homeostasis | 11 | 8.46154 | 0.010 | *MCUR1, SCO1, CHP1, TNNI3, TRPM8, ATP6V0E2, GPR89B, SLC39A4, HSP90B1, TRPM4, SNCA* |
| GOTERM_BP_FAT | GO:0055114~oxidation-reduction process | 14 | 10.7692 | 0.010 | *CYP2J2, ABCC4, SMOX, DCXR, SORD, ACAT2, PDIA5, C15ORF48, ALDH1A3, RDH11, EHHADH, CRYL1, DHCR7, SNCA* |
| GOTERM_BP_FAT | GO:0071310~cellular response to organic substance | 24 | 18.4615 | 0.011 | *SPON2, XBP1, PAQR6, PLA2G4F, GALNT3, STAT1, IFI6, ISG15, WIPI1, MIF, FGFRL1, PDIA5, GNAI1, HIPK2, HSP90B1, KRT18, SERP1, OAS2, OAS3, DNAJB9, ATP6V0E2, IRF9, TRPM4, SNCA* |
| GOTERM_BP_FAT | GO:0005975~carbohydrate metabolic process | 12 | 9.23077 | 0.011 | *SERP1, GALNT3, OAS2, DCXR, RPN1, TFF3, SORD, CHP1, CTBS, GNE, MPDU1, SNCA* |
| GOTERM_BP_FAT | GO:0006633~fatty acid biosynthetic process | 5 | 3.84615 | 0.014 | *XBP1, PLA2G4F, ELOVL5, PCCB, MIF* |
| GOTERM_BP_FAT | GO:0060333~interferon-gamma-mediated signaling pathway | 4 | 3.07692 | 0.015 | *STAT1, OAS2, OAS3, IRF9* |
| GOTERM_BP_FAT | GO:0019585~glucuronate metabolic process | 3 | 2.30769 | 0.015 | *DCXR, SORD, CRYL1* |
| GOTERM_BP_FAT | GO:0006063~uronic acid metabolic process | 3 | 2.30769 | 0.015 | *DCXR, SORD, CRYL1* |
| GOTERM_BP_FAT | GO:0051607~defense response to virus | 6 | 4.61538 | 0.016 | *SPON2, STAT1, OAS2, OAS3, ISG15, IRF9* |
| GOTERM_BP_FAT | GO:1901615~organic hydroxy compound metabolic process | 8 | 6.15385 | 0.017 | *SEC14L2, INPP4B, ALDH1A3, PSAT1, RDH11, SORD, DHCR7, SNCA* |
| GOTERM_BP_FAT | GO:0048878~chemical homeostasis | 14 | 10.7692 | 0.017 | *XBP1, MCUR1, STAT1, IFI6, GPR89B, HSP90B1, SCO1, CHP1, TNNI3, TRPM8, ATP6V0E2, SLC39A4, TRPM4, SNCA* |
| GOTERM_BP_FAT | GO:0042592~homeostatic process | 19 | 14.6154 | 0.017 | *XBP1, MCUR1, STAT1, IFI6, ISG15, MIF, GPR89B, PDIA5, HIPK2, HSP90B1, SCO1, CHP1, TNNI3, POTEF, TRPM8, ATP6V0E2, SLC39A4, TRPM4, SNCA* |
| GOTERM_BP_FAT | GO:0045087~innate immune response | 12 | 9.23077 | 0.018 | *SPON2, RPS6KA3, STAT1, OAS2, OAS3, IFI6, RAB27A, ISG15, MIF, IRF9, HSP90B1, SNCA* |
| GOTERM_BP_FAT | GO:0008610~lipid biosynthetic process | 10 | 7.69231 | 0.019 | *SEC14L2, INPP4B, ALDH1A3, XBP1, PLA2G4F, ELOVL5, PCCB, MBOAT2, DHCR7, MIF* |
| GOTERM_BP_FAT | GO:1902930~regulation of alcohol biosynthetic process | 3 | 2.30769 | 0.020 | *SEC14L2, DHCR7, SNCA* |
| GOTERM_BP_FAT | GO:0034341~response to interferon-gamma | 5 | 3.84615 | 0.020 | *STAT1, OAS2, OAS3, IRF9, SNCA* |
| GOTERM_BP_FAT | GO:1901137~carbohydrate derivative biosynthetic process | 11 | 8.46154 | 0.024 | *SERP1, GALNT3, OAS2, RFK, DCXR, RPN1, SORD, CRYL1, CHP1, GNE, MPDU1* |
| GOTERM_BP_FAT | GO:0044723~single-organism carbohydrate metabolic process | 10 | 7.69231 | 0.025 | *SERP1, GALNT3, OAS2, DCXR, RPN1, SORD, CHP1, CTBS, MPDU1, SNCA* |

| | | | | | |
|---|---|---|---|---|---|
| GOTERM_BP_FAT | GO:0070887~cellular response to chemical stimulus | 26 | 20 | 0.028 | *SPON2, IFI6, FGFRL1, GNAI1, HSP90B1, SERP1, DNAJB9, ATP6V0E2, TRPM4, SNCA, CYP2J2, XBP1, PAQR6, PLA2G4F, GALNT3, STAT1, WIPI1, ISG15, MIF, PDIA5, HIPK2, KRT18, OAS2, OAS3, SAA2, IRF9* |
| GOTERM_BP_FAT | GO:1901135~carbohydrate derivative metabolic process | 15 | 11.5385 | 0.029 | *GALNT3, DCXR, RPN1, SORD, SULT2B1, SERP1, OAS2, RFK, CRYL1, CHP1, CTBS, PDE9A, GNE, MPDU1, SNCA* |
| GOTERM_BP_FAT | GO:0034976~response to endoplasmic reticulum stress | 6 | 4.61538 | 0.030 | *XBP1, SERP1, DNAJB9, WIPI1, PDIA5, HSP90B1* |
| GOTERM_BP_FAT | GO:0090407~organophosphate biosynthetic process | 9 | 6.92308 | 0.031 | *INPP4B, XBP1, OAS2, RFK, DCXR, SORD, MBOAT2, CRYL1, SNCA* |
| GOTERM_BP_FAT | GO:0002700~regulation of production of molecular mediator of immune response | 4 | 3.07692 | 0.032 | *SPON2, XBP1, MIF, TRPM4* |
| GOTERM_BP_FAT | GO:0032309~icosanoid secretion | 3 | 2.30769 | 0.032 | *ABCC4, PLA2G4F, MIF* |
| GOTERM_BP_FAT | GO:0010033~response to organic substance | 27 | 20.7692 | 0.033 | *SPON2, IFI6, FGFRL1, GNAI1, HSP90B1, RPS6KA3, SERP1, DNAJB9, ATP6V0E2, TRPM4, SNCA, ABCC4, XBP1, PAQR6, PLA2G4F, GALNT3, STAT1, SORD, WIPI1, ISG15, MIF, PDIA5, HIPK2, KRT18, OAS2, OAS3, IRF9* |
| GOTERM_BP_FAT | GO:0033559~unsaturated fatty acid metabolic process | 4 | 3.07692 | 0.038 | *CYP2J2, PLA2G4F, ELOVL5, MIF* |
| GOTERM_BP_FAT | GO:0071715~icosanoid transport | 3 | 2.30769 | 0.038 | *ABCC4, PLA2G4F, MIF* |
| GOTERM_BP_FAT | GO:0006875~cellular metal ion homeostasis | 8 | 6.15385 | 0.039 | *MCUR1, SCO1, TNNI3, TRPM8, SLC39A4, HSP90B1, TRPM4, SNCA* |
| GOTERM_BP_FAT | GO:0009605~response to external stimulus | 21 | 16.1538 | 0.040 | *SPON2, CNTNAP2, XBP1, STAT1, PLEKHB1, SORD, ISG15, MIF, TTN, RPS6KA3, ALDH1A3, RELN, RDH11, OAS2, OAS3, SAA2, TRPM8, IRF9, SLC39A4, TRPM4, SNCA* |
| GOTERM_BP_FAT | GO:0009581~detection of external stimulus | 4 | 3.07692 | 0.040 | *RDH11, PLEKHB1, TRPM8, TTN* |
| GOTERM_BP_FAT | GO:0009582~detection of abiotic stimulus | 4 | 3.07692 | 0.043 | *RDH11, PLEKHB1, TRPM8, TTN* |
| GOTERM_BP_FAT | GO:0008215~spermine metabolic process | 2 | 1.53846 | 0.044 | *SMOX, SMS* |
| GOTERM_BP_FAT | GO:0002252~immune effector process | 10 | 7.69231 | 0.049 | *SPON2, XBP1, STAT1, OAS2, OAS3, RAB27A, ISG15, MIF, IRF9, TRPM4* |
